# Supplementary figures and images for: Cyclocytidine hydrochloride inhibits the synthesis of relaxed circular DNA of hepatitis B virus
Source: PeerJ. 2022 Jul 12;10:e13719. doi: 10.7717/peerj.13719 (PMC9285472; doi:10.7717/peerj.13719)

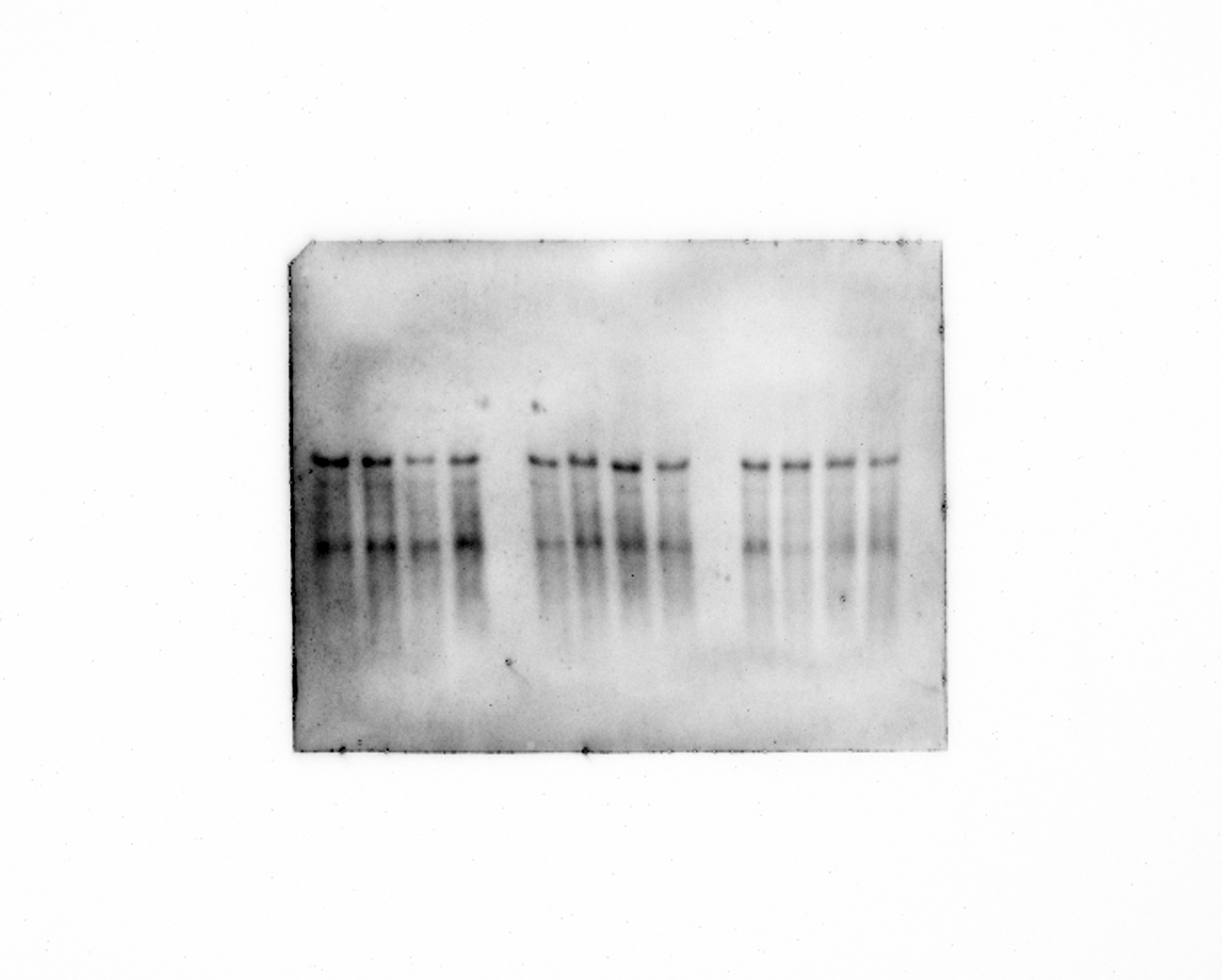

Supplement: Supplemental Information 1 [file peerj-10-13719-s001.tif]

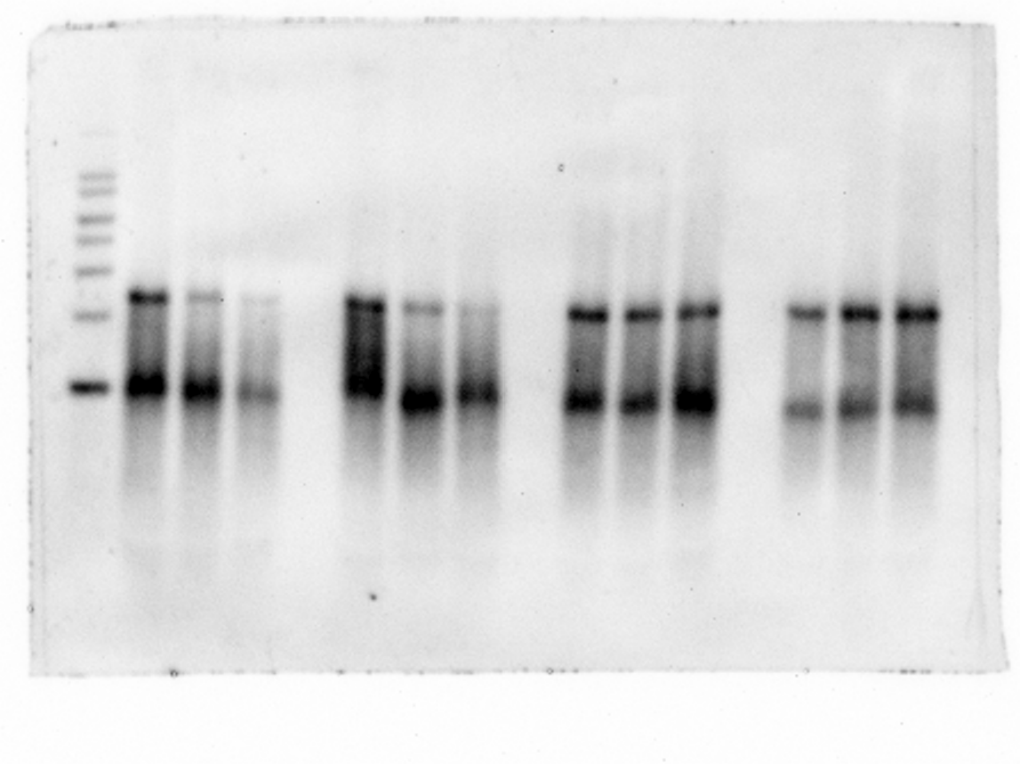

Supplement: Supplemental Information 2 [file peerj-10-13719-s002.tif]

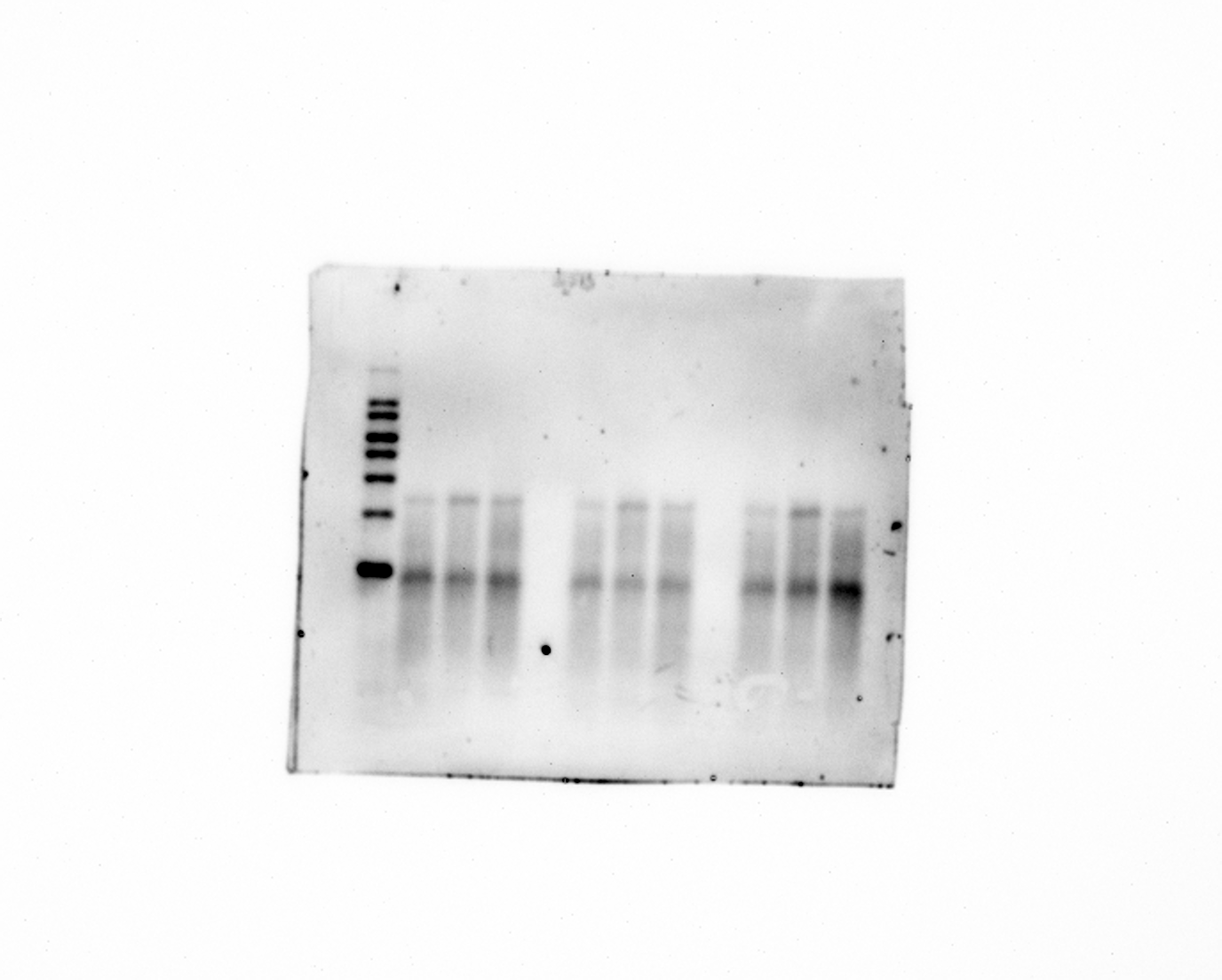

Supplement: Supplemental Information 3 [file peerj-10-13719-s003.tif]

**A.**

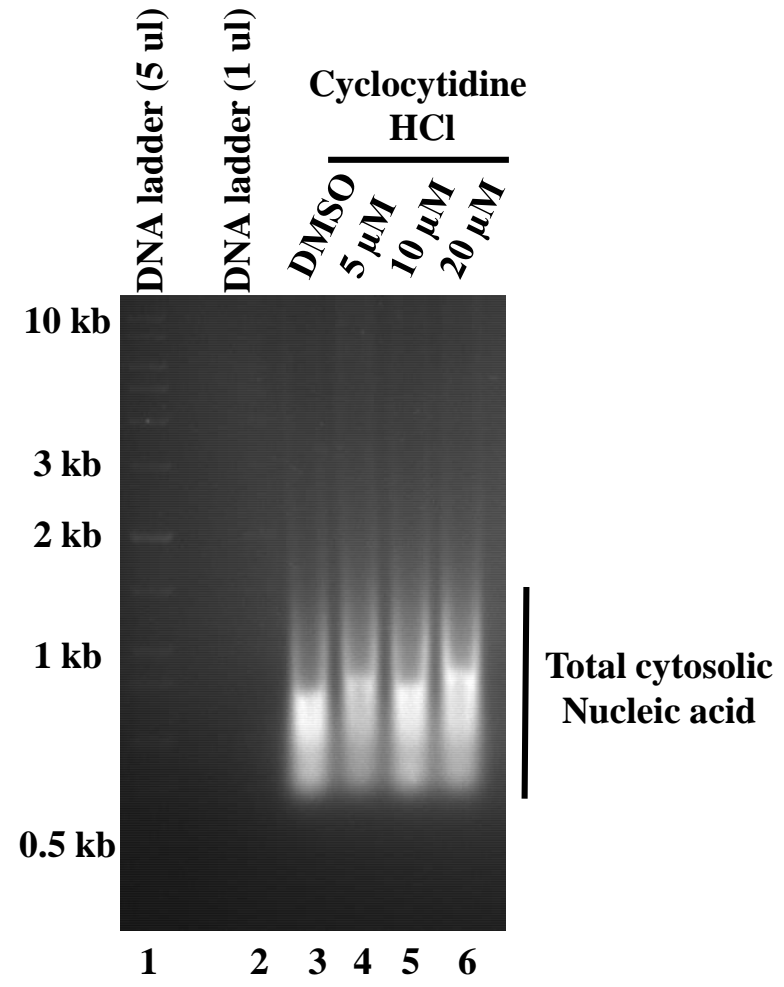

**B.**

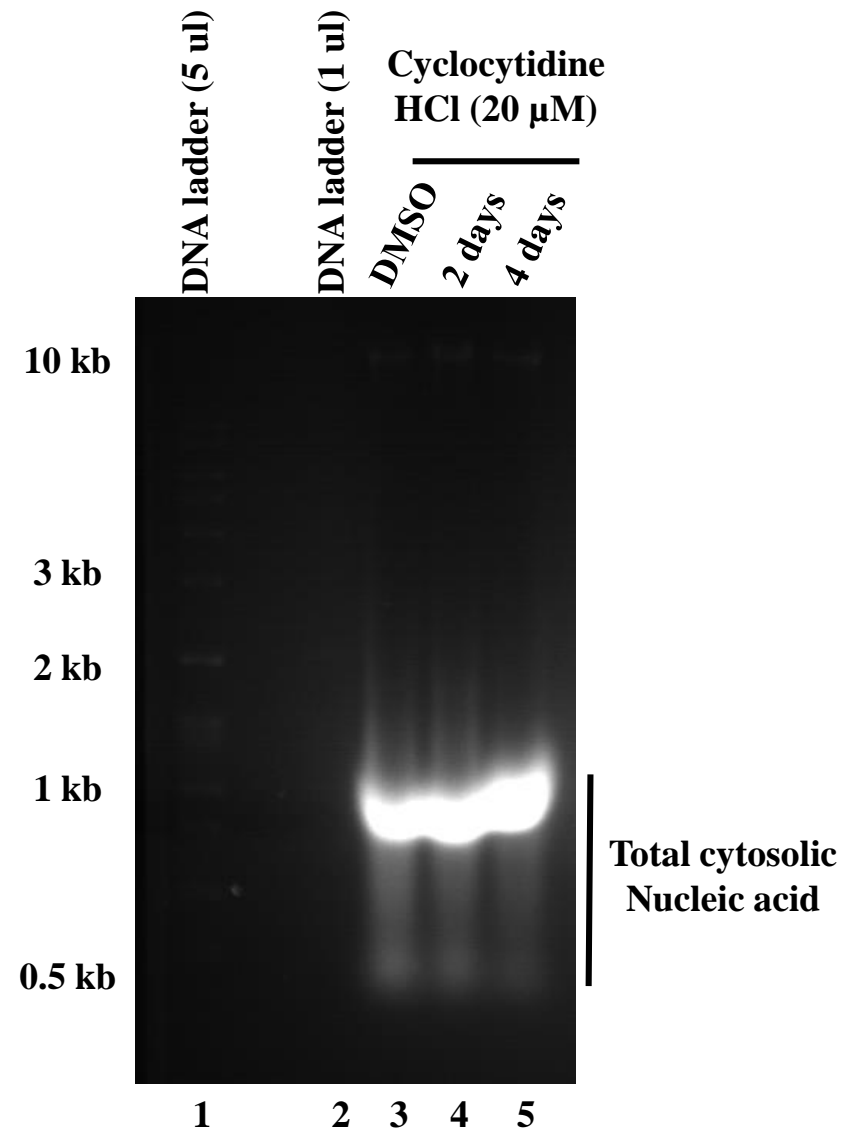

Supplement: Supplemental Information 4 — After finishment of agarose gel electrophoresis, the cytosolic nucleic acid in the gel was visiualized under ultroviolate light and the image was taken by gel imaging system. the densitometry data of nucleic acid was measeured by ImageJ software and exibited as bar chart. [file peerj-10-13719-s004.pdf]

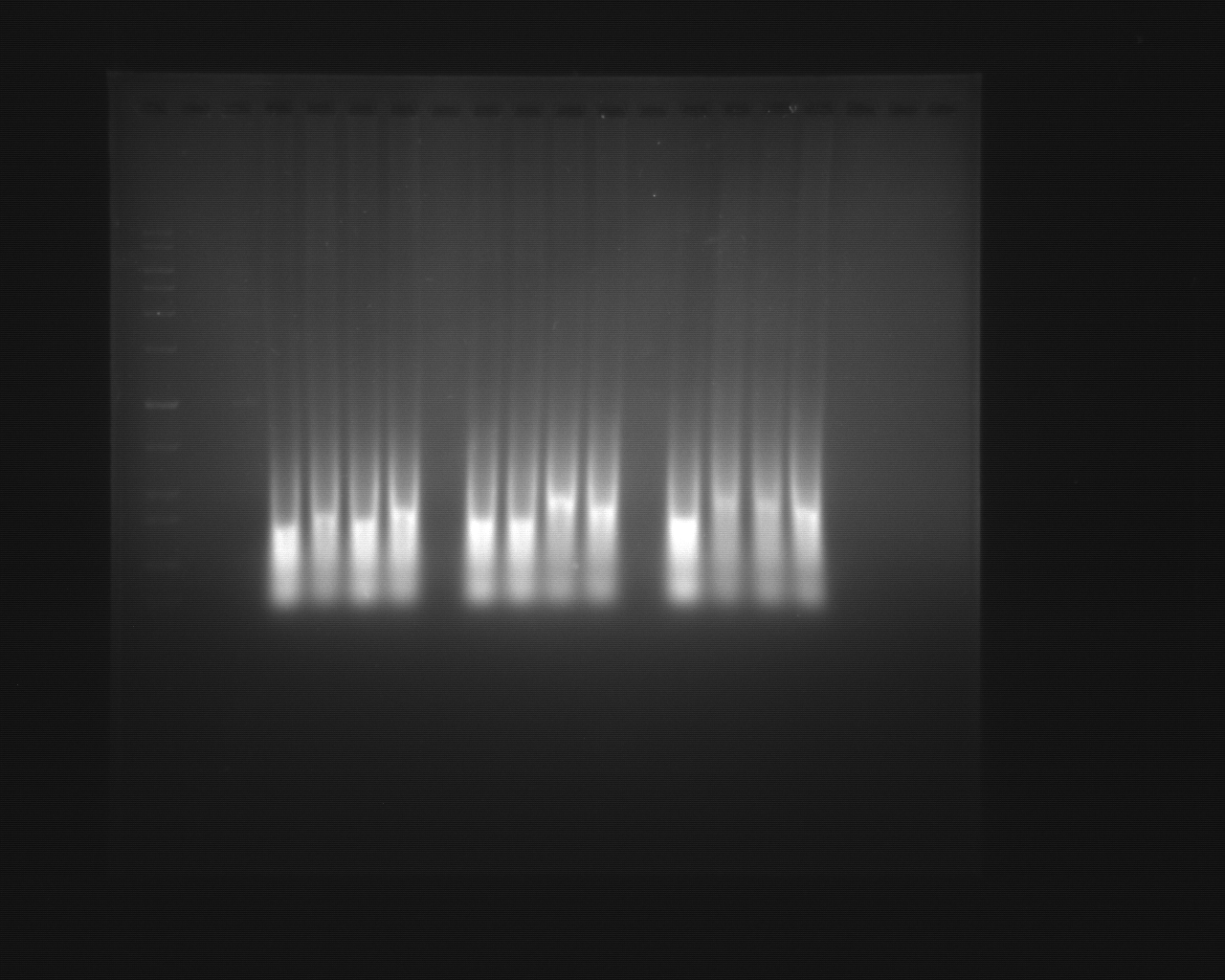

Supplement: Supplemental Information 5 [file peerj-10-13719-s005.tif]

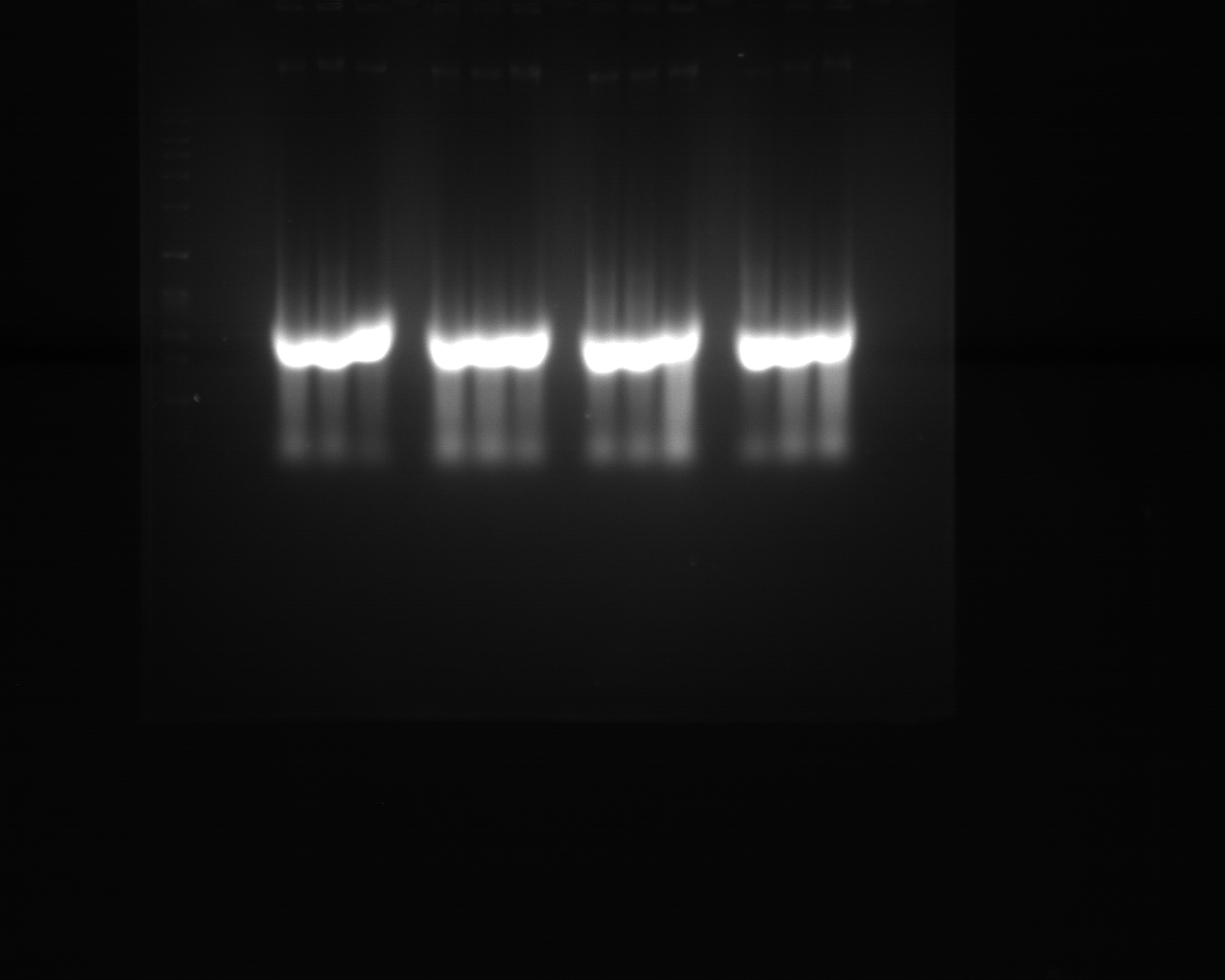

Supplement: Supplemental Information 6 [file peerj-10-13719-s006.tif]
