# Supplementary material for: Cyclocytidine hydrochloride inhibits the synthesis of relaxed circular DNA of hepatitis B virus
Source: PeerJ. 2022 Jul 12;10:e13719. doi: 10.7717/peerj.13719 (PMC9285472; doi:10.7717/peerj.13719)
Supplement: Supplemental Information 12 [file peerj-10-13719-s012.pdf]

HBV DNA, genotype D, 3182 bp

CCACAACCTTTCACCAAACTCTGCAAGATCCCAGAGTGAGAGGCCTGTATTTCCCTGC
TGGTGGCTCCAGTTCAGGAGCAGTAAACCCTGTTCCGACTACTGCCTCTCCCTTATCGT
CAATCTTCTCGAGGATTGGGGACCCTGCGCTGAACATGGAGAACATCACATCAGGATT
CCTAGGACCCCTTCTCGTGTTACAGGCGGGGTTTTTCTTGTTGACAAGAATCCTCACAA
TACCGCAGAGTCTAGACTCGTGGTGGACTTCTCTCAATTTTCTAGGGGGAACTACCGTG
TGTCTTGGCCAAAATTCGCAGTCCCCAACCTCCAATCACTCACCAACCTCCTGTCCTCC
AACTTGTCCTGGTTATCGCTGGATGTGTCTGCGGCGTTTTATCATCTTCCTCTTCATCCT
GCTGCTATGCCTCATCTTCTTGTTGGTTCTTCTGGACTATCAAGGTATGTTGCCCGTTTG
TCCTCTAATTCCAGGATCCTCAACCACCAGCACGGGACCATGCCGAACCTGCATGACT
ACTGCTCAAGGAACCTCTATGTATCCCTCCTGTTGCTGTACCAAACCTTCGGACGGAAA
TTGCACCTGTATTCCCATCCCATCATCCTGGGCTTTCGGAAAATTCCTATGGGAGTGGG
CCTCAGCCCGTTTCTCCTGGCTCAGTTTACTAGTGCCATTTGTTCAGTGGTTCGTAGGG
CTTTCCCCCACTGTTTGGCTTTCAGTTATATGGATGATGTGGTATTGGGGGCCAAGTCTG
TACAGCATCTTGAGTCCCTTTTTACCGCTGTTACCAATTTTCTTTTGTCTTTGGGTATACA
TTTAAACCCTAACAAAACAAAGAGATGGGGTTACTCTCTGAATTTTATGGGTTATGTCA
TTGGAAGTTATGGGTCCTTGCCACAAGAACACATCATACAAAAAATCAAAGAATGTTT
TAGAAAACTTCCTATTAACAGGCCTATTGATTGGAAAGTATGTCAACGAATTGTGGGTC
TTTTGGGTTTTGCTGCCCCATTTACACAATGTGGTT
ATCCTGCGTTAATGCCCTTGTATGCATGTATTCAATCTAAGCAGGCTTTCACTTTCTCGC
CAACTTACAAGGCCTTTCTGTGTAAACAATACCTGAACCTTTACCCCGTTGCCCGGCAA
CGGCCAGGTCTGTGCCAAGTGTTTGCTGACGCAACCCCCACTGGCTGGGGCTTGGTCA
TGGGCCATCAGCGCGTGCGTGGAACCTTTTCGGCTCCTCTGCCGATCCATACTGCGGA
ACTCCTAGCCGCTTGTTTTGCTCGCAGCAGGTCTGGAGCAAACATTATCGGGACTGATA
ACTCTGTTGTCCTCTCCCGCAAATATACATCGTATCCATGGCTGCTAGGCTGTGCTGCCA
ACTGGATCCTGCGCGGGACGTCCTTTGTTTACGTCCCGTCGGCGCTGAATCCTGCGGA
CGACCCTTCTCGGGGTCGCTTGGGACTCTCTCGTCCCCTTCTCCGTCTGCCGTTCCGAC
CGACCACGGGGCGCACCTCTCTTTACGCGGACTCCCCGTCTGTGCCTTCTCATCTGCCG
GACCGTGTGCACTTCGCTTCACCTCTGCACGTCGCATGGAGACCACCGTGAACGCCCA
CCGAATGTTGCCCAAGGTCTTACATAAGAGGACTCTTGGACTCTCTGCAATGTCAACG
ACCGACCTTGAGGCATACTTCAAAGACTGTTTGTTTAAAGACTGGGAGGAGTTGGGGG
AGGAGATTAGATTAAAGGTCTTTGTACTAGGAGGCTGTAGGCATAAATTGGTCTGCGCA
CCAGCACCATGCAACTTTTTCACCTCTGCCTAATCATCTCTTGTTCATGTCCTACTGTTC
AAGCCTCCAAGCTGTGCCTTGGGTGGCTTTGGGGCATGGACATCGACCCTTATAAAGA
ATTTGGAGCTACTGTGGAGTTACTCTCGTTTTTGCCTTCTGACTTCTTTCCTTCAGTACG
AGATCTTCTAGATACCGCCTCAGCTCTGTATCGGGAAGCCTTAGAGTCTCCTGAGCATT
GTTCACCTCACCATACTGCACTCAGGCAAGCAATTCTTTGCTGGGGGGAACTAATGAC
TCTAGCTACCTGGGTGGGTGTTAATTTGGAAGATCCAGCATCTAGAGACCTAGTAGTCA
GTTATGTCAACACTAATATGGGCCTAAAGTTCAGGCAACTCTTGTGGTTTCACATTTCTT
GTCTCACTTTTGGAAGAGAAACCGTTATAGAGTATTTGGTGTCTTTCGGAGTGTGGATT
CGCACTCCTCCAGCTTATAGACCACCAAATGCCCCTATCCTATCAACACTTCCGGAAAC
TACTGTTGTTAGACGACGAGGCAGGTCCCCTAGAAGAAGAACTCCCTCGCCTCGCAGA
CGAAGGTCTCAATCGCCGCGTCGCAGAAGATCTCAATCTCGGGAACCTCAATGTTAGT

ATTCCTTGGACTCATAAGGTGGGGAACTTTACTGGTCTTTATTCTTCTACTGTACCTGTC
TTTAATCCTCATTGGAAAACACCATCTTTTCCTAATATACATTTACACCAAGACATTATCA
AAAAATGTGAACAGTTTGTAGGCCCACTTACAGTTAATGAGAAAAGAAGATTGCAATT
GATTATGCCTGCTAGGTTTTATCCAAAGGTTACCAAATATTTACCATTGGATAAGGGTAT
TAAACCTTATTATCCAGAACATCTAGTTAATCATTACTTCCAAACTAGACACTATTTACA
CACTCTATGGAAGGCGGGTATATTATATAAGAGAGAAACAACACATAGCGCCTCATTTT
GTGGGTCACCATATTCTTGGGAACAAGATCTACAGCATGGGGCAGAATCTTTCCACCA
GCAATCCTCTGGGATTCTTTCCCGACCACCAGTTGGATCCAGCCTTCAGAGCAAACAC
AGCAAATCCAGATTGGGACTTCAATCCCAACAAGGACACCTGGCCAGACGCCAACAA
GGTAGGAGCTGGAGCATTCGGGCTGGGTTTCACCCCACCGCACGGAGGCCTTTTGGGG
TGGAGCCCTCAGGCTCAGGGCATACTACAAACTTTGCCAGCAAATCCGCCTCCTGCCT
CCACCAATCGCCAGACAGGAAGGCAGCCTACCCCGCTGTCTCCACCTTTGAGAAACAC
TCATCCTCAGGCCATGCAGTGGAATT
